# Vitamin D receptor genetic polymorphisms are associated with oral lichen planus susceptibility in a Chinese Han population

**DOI:** 10.1186/s12903-020-1002-3

**Published:** 2020-01-30

**Authors:** Hong Shen, Qinglan Liu, Peng Huang, Haozhi Fan, Feng Zang, Mei Liu, Lingyun Zhuo, Jingjing Wu, Guoying Wu, Rongbin Yu, Jianrong Yang

**Affiliations:** 1 Department of Pediatric and Preventive dentistry, Affiliated Hospital of Stomatology, Nanjing Medical University, Jiangsu Key Laboratory of Oral Diseases, Nanjing, 210029 China; 2 Department of oral mucosal disease, Affiliated Hospital of Stomatology, Nanjing Medical University, Jiangsu Key Laboratory of Oral Diseases, Nanjing, 210029 China; 3 Department of Epidemiology and Biostatistics, School of Public Health, Key Laboratory of Infectious Diseases, Nanjing Medical University, Nanjing, 211166 China; 4 Department of Oral and Maxillofacial Surgery, Affiliated Hospital of Stomatology, Nanjing Medical University, Jiangsu Key Laboratory of Oral Diseases, Nanjing, 210029 China

**Keywords:** Oral lichen planus, Vitamin D receptors, Risk, Single nucleotide polymorphisms

## Abstract

**Background:**

Vitamin D receptor (VDR) is involved in multiple immune-mediated disorders including oral lichen planus (OLP). This study investigated the association between *VDR* gene polymorphisms and the risk of OLP.

**Methods:**

In total, 177 OLP patients and 207 healthy participants were recruited from the Affiliated Hospital of Stomatology, Nanjing Medical University. Eight single nucleotide polymorphisms (SNPs: rs731236, rs739837, rs757343, rs2107301, rs2239185, rs7975232, rs11574129 and rs11568820) in the *VDR* gene were selected and genotyped.

**Results:**

The results showed that OLP risk was increased in subjects with the rs2239185 TT genotype (Recessive model: adjusted Odd ratio(OR) = 2.68, 95% Confidence interval(CI) = 1.28–5.62, *P* = 0.009) and rs7975232 CC genotype (Recessive model: adjusted OR = 2.25, 95% CI = 1.10–4.58, *P* = 0.026). Moreover, rs2239185 and rs7975232 (*P <* 0.01) showed significant cumulative effects on OLP risk.Haplotype analysis showed that the CC haplotype (rs2239185-rs7975232) was associated with an increased risk of OLP (OR = 3.11, 95% CI = 1.42–6.83, *P* = 0.005), compared with the AC haplotype.

Conclusion: The rs2239185 and rs7975232 variants of *VDR* may influence OLP susceptibility, and *VDR* gene polymorphisms may be candidate susceptibility regions for OLP in a Chinese Han population.

## Background

Oral lichen planus (OLP) is a chronic inflammatory disease of the oral mucosa mediated by T cells, whose etiology remains unknown. OLP is characterized as dense lymphocyte infiltration and basal keratinocyte degeneration observable under a microscope [1]. OLP, the typical clinical feature of which include white stripes, can manifest as reticular, papular, plaque-like, erosive, atrophic and bullous [2, 3]. Erosive-like lesions are considered to be the most threatening condition and are characterized by pain, ranging from mild discomfort to severe onset [4].The pain seriously affects the patient’s eating experience and food digestion, reducing the patients quality of life.

Previous studies have suggested that vitamin D (VD) deficiency may be associated with an increased risk of some inflammatory diseases, such as OLP and inflammatory bowel disease [5, 6]. OLP patients presented a nearly 50% reduction in mucosal VD levels, which may be caused by immunoreactions [6]. As a ligand-induced transcription factor, vitamin D receptors (VDRs) encoded by the *VDR* gene (chromosomal location 12q12–14) play an important role in regulating the role of vitamin D [7.8]. Increasing evidence suggests that single nucleotide polymorphisms (SNPs) in vitamin D-related genes could affect the properties of vitamin D, such as its anti-carcinogenic effects [7]. Thus, we speculated that *VDR* gene polymorphisms may be related to OLP. Since OLP is considered as a potential precancerous lesion, specific SNPs in VDR or vitamin D pathway genes may also play an important role in oral cancer.

Based on the above, we conducted this study in a Chinese Han population to investigate the association between key polymorphisms in *VDR* genes and OLP susceptibility.

## Material and methods

### Study groups and samples

Assuming the control group with a mutation frequency of 0.3, OR value of 2, statistical power of 0.8 and Type I error probability of 0.05, the current study may need to recruit 141 case patients and 141 control patients to be able to reject the null hypothesis that the probability is equal to 1. A total of 177 patients with OLP from the Affiliated Hospital of Stomatology, Nanjing Medical University, Jiangsu Province, China were enrolled in this study between January 2017 and June 2018.The inclusion criteria for OLP patients were as follows: (1) > 18 years old; (2) diagnosed as OLP by an oral pathologist using a biopsy specimen; and (3) treatment-naïve (the patient did not receive relevant treatment prior to inclusion in the study). Pregnant women, patients with other oral diseases (for example, lichenoid reaction lesions), patients receiving systemic or topical steroids over the past three months, and patients with autoimmune diseases were excluded. The control group included 207 healthy subjects who underwent a physical examination at the physical examination center and had no oral mucosal lesions, inflammation, infection, or autoimmune diseases such as systemic lupus erythematosus (SLE) or rheumatoid arthritis (RA). The diagnostic criteria used in this study were diagnostic guidelines developed by Van der Meij et al. according to the World Health Organization (WHO) definition of OLP [8].

The oral mucosa of all participants was assessed by two experienced oral medicine specialists. If there was a disagreement between the two examiners, a third clinically experienced oral medicine specialist would make the judgment. The main clinical features, including clinical subtype, affected sites, the number of sites, the presence of cutaneous lesions, and the types of oral lesions and symptoms, were collected for further analyses. All subjects were informed of the purpose of the study and signed the informed consents. Information, such as demographic data, alcohol consumption habits and oral hygiene, were collected by one-to-one surveys using a questionnaire designed according to our research content. Prior to OLP diagnosis, participants who drank more than 20 alcoholic drinks per week were classified as heavy drinkers [9]. The periodontal status of all subjects including the gingival index (GI), periodontal index (PI) and bleeding on probing (BOP) was evaluated in both groups. The oral hygiene of subjects was defined as poor when both GI and PI were ≥ 2, and the BOP was 1. Additionally, 10 ml of venous blood was collected from each subject for biochemical tests and SNP determination.

### DNA isolation and genotyping

Genomic DNA was extracted from peripheral blood samples using protease K digestion and phenol/chloroform purification according to a standard protocol. The TaqMan allelic discrimination technology via an ABI 7900HT Sequence Detection system (Applied Biosystems, San Diego, California, USA) was used to explore polymorphisms at the chosen SNPs. Polymerase chain reaction (PCR) was executed with the following thermal profile: 50 twarer 2 min to preheat, 95 °C for 10 min for preincubate, followed by 40 cycles at 95 e with the 50 twarer 2 min to anneal thermal profile. The genotyping results were detected by LightCycler480 real-time PCR (Roche Diagnostics, Mannheim, Germany), with a 100% success rate. Two blank controls were specified to a 384-well format for quality control with 10% of the samples randomly selected as repeat samples, producing 100% concordance.

Information regarding *VDR* SNPs was acquired from the NCBI dbSNP database (http://www.ncbi.nlm.nih.gov/SNP) and the Chinese Han population database in HapMap (http://www.hapmap.org). All SNPs were selected according to the following criteria: (1) minor allele frequency (MAF) ≥0.05 in the Chinese population and (2) a Hardy-Weinberg equilibrium test *P-*value ≥0.05. Tag SNPs were chosen to represent a set of variants with strong linkage disequilibrium (LD) [9]. A total of eight SNPs in the *VDR* gene (rs731236, rs739837, rs757343, rs2107301, rs2239185, rs7975232, rs11574129 and rs11568820) were selected according to the above steps.

### Statistical analysis

All analyses were performed in Stata/SE (V.12.0 for Windows). Differences in the demographic characteristics between the case and control groups were analyzed by the *Student-t* test (for continuous variables) or chi-square (*χ*^*2*^) test (for categorical variables). The relationship between a candidate SNP and OLP risk was estimated by multivariate logistic regression analysis, and the results were expressed as OR and its 95% CI. The heterogeneity between the corresponding subgroups was examined by the *Q* test. The Cochran-Armitage test was used for trend analysis. Haplotype analysis was performed to explore the relationship between two significant SNPs and OLP risks. The PHASE software (v2.1) was used to estimate the haplotype frequency based on the observed genotypes. The Single-fold view software (version 4.2) was used to analyze LD parameters (i.e., D and r^2^) [10] and the Thesias software (version 3.1) was used to analyze associations of identified haplotypes in the *VDR* gene with OLP [11].

## Results

Demographic information on 177 OLP patients and 207 healthy subjects is shown in Table 1. There were similar age and gender distributions between the two groups (*P* = 0.155 and 0.091, respectively). However, compared with the control group, OLP patients had more alcohol consumption and better oral hygiene (*P* < 0.05).

**Table 1:** Characteristics of clinical data between OLP and control group

Variables	OLP	Control	*P-*value
	(*n* = 177)	(*n* = 207)	
Mean age, year	49.20 ± 14.14	48.47 ± 10.88	0.569
< 45	61 (34.46)	86 (41.55)	0.155
≥ 45	116 (65.54)	121 (58.45)	
Gender			
Male (%)	49 (27.68)	74 (35.75)	0.091
Female (%)	128 (72.32)	133 (64.25)	
Alcohol			
Heavy	11 (6.21)	2 (0.97)	0.005
Non Heavy	166 (93.79)	205 (99.03)	
Oral hygiene			
Good	131 (74.01)	119 (57.49)	0.001
Poor	46 (25.99)	88 (42.51)	

Abbreviation: *OLP* oral lichen planus

The genotype distribution of the eight SNPs in the two groups was described using dominant, recessive and additive genetic models in Table 2. The recessive genetic model computed by logistic regression analyses showed that rs2239185 and rs7975232 were significantly associated with OLP susceptibility. Patients carrying the rs2239185-TT genotype (adjusted OR = 2.39, 95%CI = 1.10–5.18, *P* = 0.027) and rs7975232-CC genotype (adjusted OR = 2.65, 95%CI = 1.24–5.66, *P* = 0.012) tended to have a higher risk of OLP.

**Table 2:** Distribution of VDR genotypes between OLP and control group

Genotype	OLP	Control	OR (95% CI)	*P-*value
rs731236				
CC	154 (87.01)	180 (86.96)	1.00	–
CT	21 (11.86)	24 (11.59)	0.99 (0.51–1.91)	0.979
TT	2 (1.13)	3 (1.45)	0.93 (0.15–6.06)	0.946
Dominant			0.99 (0.52–1.85)	0.965
Recessive			0.94 (0.15–6.05)	0.947
Additive			0.98 (0.57–1.71)	0.954
rs739837				
CC	98 (55.37)	112 (54.11)	1.00	–
CA	61 (34.46)	82 (39.61)	0.73 (0.45–1.20)	0.218
AA	18 (10.17)	13 (6.28)	1.36 (0.60–3.09)	0.468
Dominant			0.82 (0.51–1.31)	0.404
Recessive			1.60 (0.73–3.50)	0.242
Additive			0.98 (0.69–1.41)	0.927
rs757343				
AA	113 (63.84)	128 (61.84)	1.00	–
AG	53 (29.94)	73 (35.27)	0.70 (0.43–1.15)	0.163
GG	11 (6.21)	6 (2.90)	2.06 (0.70–6.13)	0.180
Dominant			0.81(0.50–1.29)	0.375
Recessive			2.36 (0.81–6.89)	0.117
Additive			0.97 (0.66–1.44)	0.893
rs2107301				
CC	83 (46.89)	96 (46.38)	1.00	–
CT	79 (44.63)	93 (44.93)	1.03 (0.67–1.60)	0.883
TT	15 (8.47)	18 (8.70)	1.19 (0.54–2.58)	0.668
Dominant			1.06 (0.69–1.61)	0.800
Recessive			1.17 (0.55–2.45)	0.686
Additive			1.07 (0.77–1.48)	0.706
rs2239185				
CC	90 (50.85)	111 (53.62)	1.00	–
CT	62 (35.03)	83 (40.10)	0.78 (0.47–1.27)	0.315
TT	25 (14.12)	13 (6.28)	2.39 (1.10–5.18)	0.027
Dominant			1.00 (0.63–1.59)	0.995
Recessive			2.68 (1.28–5.62)	0.009
Additive			1.24 (0.88–1.74)	0.206
rs7975232				
AA	81 (45.76)	118 (57.00)	1.00	–
AC	70 (39.55)	74 (35.75)	1.36 (0.83–2.23)	0.225
CC	26 (14.69)	15 (7.25)	2.65 (1.24–5.66)	0.012
Dominant			1.57 (0.97–2.50)	0.061
Recessive			2.25 (1.10–4.58)	0.026
Additive			1.54 (1.09–2.18)	0.014
rs11574129				
CC	119 (67.23)	146 (70.53)	1.00	–
CT	50 (28.25)	57 (27.54)	0.97 (0.59–1.61)	0.921
TT	8 (4.52)	4 (1.93)	2.49 (0.70–8.84)	0.159
Dominant			1.08 (0.67–1.75)	0.740
Recessive			2.50 (0.71–8.84)	0.154
Additive			1.17 (0.78–1.76)	0.439
rs11568820				
AA	61 (34.46)	74 (35.75)	1.00	–
AG	81 (45.76)	96 (46.38)	1.10 (0.69–1.76)	0.695
GG	35 (19.77)	37 (17.87)	1.26 (0.69–2.28)	0.454
Dominant			1.14 (0.74–1.78)	0.551
Recessive			1.19 (0.70–2.03)	0.520
Additive			1.12 (0.83–1.50)	0.455

Logistic regression analyses adjusted for age, gender, alcohol, oral hygiene

Abbreviation:*VDR* vitamin D receptor, *OLP* oral lichen planus, *OR* odds ratio, *CI* confident interval

The cumulative effects of the two SNPs on OLP were evaluated by the Cochran-Armitage trend test. The results showed that the risk of OLP increased as the number of mutations increased (Table 3). The OLP risk increased in subjects carrying one or both of the rs2239185 and rs7975232 alleles (OR = 2.33, 95% CI = 1.22–4.43). In stratified analyses on the combined variant alleles (rs2239185 and rs7975232) and OLP risk susceptibility, no heterogeneity was observed (Table 4).

**Table 3:** Cumulative Effects of rs2239185 and rs7975232 on OLP risk

Variables	OLP n(%)	Control n(%)	OR(95%CI)	*P-*value
0	145 (81.92)	188 (90.82)	1.00	–
1	13 (7.34)	10 (4.83)	1.82 (0.74–4.48)	0.190
2	19 (10.73)	9 (4.35)	2.87 (1.21–6.81)	0.017^*^
Trend				*P* ^a^ = 0.007
0	145 (81.92)	188 (90.82)	1.00	–
1–2	32 (18.08)	19 (9.18)	2.33 (1.22–4.43)	0.010

Variables are numbers of combined unfavorable alleles (rs2239185-TT and rs7975232-CC)

Logistic regression analyses adjusted for age, gender, alcohol consumption and oral hygiene. *P*^*a*^*-*value was analyzed by Cochran-Armitage trend test

Abbreviation: *OLP* oral lichen planus, *OR* odds ratio, *CI* confident interval

**Table 4:** Stratified analyses on combined variant alleles (rs2239185 and rs7975232) and OLP risk

Variables	OLP risk (0 vs.1–2)		OR (95%CI)	*P* ^*a*^	*P* ^*b*^
	OLP	Control			
Age, year					
≥ 45	50/11	75/11	1.69 (0.60–4.71)	0.318	0.457
< 45	95/21	133/8	3.13 (1.28–7.65)	0.012	
Gender					
Male	40/9	58/16	0.61 (0.21–1.72)	0.347	0.267
Female	105/23	130/3	10.11 (2.82–36.30)	< 0.001	
Alcohol					
Heavy	8/3	1/1	–	–	–
Non Heavy	137/29	187/18	2.42 (1.25–4.67)	0.008	
Oral hygiene					
Good	109/22	103/16	1.59 (0.75–3.37)	0.231	0.453
Poor	36/10	85/3	6.47 (1.56–26.90)	0.010	

Logistic regression was used in the implicit model to determine the adjusted *P* ^*a*^ value according to age, gender, alcohol consumption and oral hygiene, heterogeneity was used to test

*P* ^*b*^*-*value

Abbreviation: *OLP* oral lichen planus, *OR* odds ratio, *CI* confident interval

LD information for the two SNPs was shown in Table 5. We performed haplotype analysis to assess the effects of the rs2239185 and rs7975232 haplotype variant alleles on the OLP risk (Table 5). When compared with the most frequent AC haplotype, the CC haplotype was significantly associated with OLP susceptibility (OR = 3.11, 95%CI = 1.42–6.83), which was consistent with the single SNP analysis.

**Table 5:** Haplotypes analysis of rs2239185 and rs7975232 with OLP risk

Haplotype	OLP *n*(%)	Control *n*(%)	OR	*P*
AC	218 (61.59)	295 (71.26)	1.00	1.00
CT	98 (27.68)	94 (22.70)	1.38 (0.96–1.97)	0.084
CC	24 (6.78)	10 (2.42)	3.11 (1.42–6.83)	0.005
AT	14 (3.95)	15 (3.62)	1.21 (0.55–2.66)	0.633

Logistic regression analyses adjusted for age, gender,alcohol consumption and oral hygiene

SNPs order: rs2239185 and rs797523

**Fig. 1:**
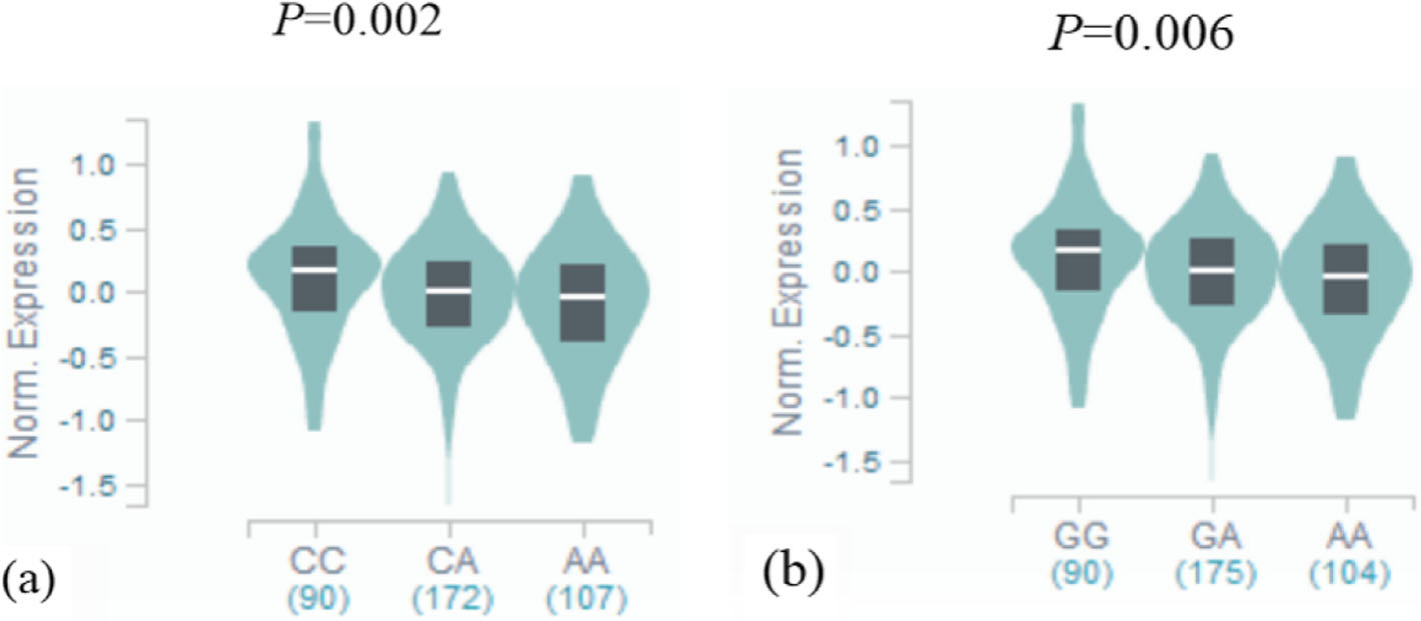
**a** Results of eQTL analysis on *VDR* rs7975232 loci. in whole blood. **b**. Results of eQTL analysis on *VDR* rs2239185 loci. in whole blood

To further explore the biological significance of rs2239185 and rs7975232 in *VDR*, we also searched for expression quantitative trait loci (eQTL) evidence based on the public Genotype-Tissue Expression(GTEx) database (https://gtexportal.org/). We found that the *VDR* rs2239185 and rs7975232 genotypes were significantly associated with the expression of VDR in whole blood. Mutations associated with VDR rs7975232 and rs2239185 down-regulated of *VDR* gene expression in whole blood (*P* = 0.002 and 0.006, respectively, Fig. 1).

## Discussion

OLP is one of the main causes of oral cancer and approximately 0.5 to 2.0% of patients with OLP can progress to malignant transformation [12]. Finding new markers that can effectively identify people at a high risk for OLP can help prevent and control OLP, thereby reducing the progression of malignant transformation. Previous studies have confirmed the role of multiple factors including genetic polymorphisms in OLP susceptibility [13]. The current study aimed to further explore the effects of genetic variation in the *VDR* gene and related environmental factors on OLP susceptibility in Chinese Han population and the results showed that *VDR* rs2239185-TT and rs7975232-CC increased the susceptibility to OLP, after adjusting for factors such as drinking and oral hygiene [14, 15].

Vitamin D and its receptors are mainly involved in immune regulation and calcium and phosphorus metabolism regulation. Evidence from population studies suggests that vitamin D deficiency is highly prevalent among the general population in China, which may be related to genetic variation in *VDR* [16]. Previous studies have shown that *VDR* genetic variants (such as rs1544410, rs7975232 and rs731236) can participate in the development of various tumors (such as prostate cancer and hepatocellular carcinoma) by affecting the biological effects of vitamin D [17, 18]. Shortly thereafter, vitamin D deficiency was found to be associated to immune-related diseases (systemic lupus erythematosus) and infectious diseases (tuberculosis) [19]. On this basis, our study found the association between the genetic variation of VDR and OLP susceptibility, which further confirmed the findings of Zhao Bin et al. And their study found that the *VD/VDR* signaling pathway could prevent OLP development by mediating the NF-κB pathway [20]. The current results revealed that mutations in *VDR* rs2239185 and rs7975232 increased the risk of OLP, and OLP risk increased with increasing adverse alleles. The heterogeneity test suggested that our final results were robust and unaffected by other factors. Additionally, due to the strong LD between VDR rs7975232 and rs2239185, haplotype analysis was further conducted with the results suggested that SNP-SNP interactions in the *VDR* gene increased susceptibility to OLP, which further validated the single-gene analysis results [21–23].

The gene encoding *VDR*, which is located on chromosome 12q13, consists of 9 exons and 8 introns, and is approximately 75 kb in length [24]. Both rs2239185 and rs7975232 are located in a *VDR* gene intron, and their polymorphism can affect messenger RNA transcription and protein translation by regulating gene transcription to change the expression and function of VDR [25]. Moreover, the GTEx database showed that VDR expression in the blood was indeed affected by genetic variation associated with rs2239185 and rs7975232. Therefore, we hypothesized that the rs2239185 and rs7975232 variants, as well as the CC haplotypes between the two, may affect VDR expression in whole blood by interfering with transcription. The binding of VD to VDR in whole blood and oral keratinocytes was reduced, preventing the activation of the vitamin D/VDR signaling pathway [20], thereby inhibiting functions related to the VDR signaling pathway, such as anti-inflammatory effects, and eventually led to the occurrence of OLP.

It is worth noting that our findings differ from those of Bojan Kujundzic et al., which found that the genetic polymorphism associated with OLP risk was not *VDR* rs7975232 but *VDR* rs2228570 (rs2239185 was not included in the study) [9]. And the haplotype analysis involved rs7975232 and rs731236 suggested that the AT haplotype decreased OLP risk (compared with the most common haplotype CT), further confirming that OLP susceptibility was not caused by a single SNP. Many previous epidemiological studies have shown that the incidence of OLP, clinical symptoms and the mutation rate of *VDR* genes are different in different ethnic populations, so we speculate that this may also be the main reason for the difference between the two studies [26, 27]. The sample size for this study was smaller than our study (only 65 patients with oral lichen planus and 100 healthy blood donors), and all of study subjects were Caucasian and Serbian. Nonetheless, the results needs to be further confirmed in larger sample cohort studies and functional experimental studies.

We acknowledge that there are limitations in this study. Although various analyses were used to reveal the potential impact of *VDR* gene polymorphisms on OLP susceptibility, the existing epidemiological evidence presented in this study was not sufficient to speculate on the role of the *VDR* gene, indicating that our findings require confirmation by functional studies. However, our study also has advantages. First, this is the first study to find genetic evidence that the *VDR* gene is associated with OLP susceptibility in a Chinese Han population. Second, the sample size of this study is larger than the existing related research [9].

## Conclusion

This is the first study to show that genetic mutations in *VDR* are associated with OLP susceptibility, and that rs2239185 and rs7975232 may be the genetic markers for OLP susceptibility in a Chinese Han population. However, larger scale prospective studies and functional experiments are required to validate our findings.

## Data Availability

The data used and analysis during the current study are available from the corresponding author on reasonable request.

## Abbreviations

BOP: Bleeding on probing
CI: Confidence interval
eQTL: Expression quantitative trait loci
GI: Gingival index
GTEx: Genotype-Tissue Expression
LD: Linkage disequilibrium
MAF: Minor allele frequency
NJMU: Nanjing Medical University
OLP: Oral lichen planus
OR: Odd ratio
PCR: Polymerase chain reaction
PI: Periodontal index
RA: Rheumatoid arthritis
SLE: Systemic lupus erythematosus
SNPs: Single nucleotide polymorphisms
VD: Vitamin D
VDR: Vitamin D receptor
VDRs: Vitamin D receptors
WHO: World Health Organization
